# Determinants of associative memory performance in spiking and non-spiking neural networks with different synaptic plasticity regimes

**DOI:** 10.1186/1471-2202-13-S1-P156

**Published:** 2012-07-16

**Authors:** Alex Metaxas, Reinoud Maex, Rod Adams, Volker Steuber, Neil Davey

**Affiliations:** 1 Science and Technology Research Institute, University of Hertfordshire, Hatfield, Hertfordshire, AL10 9AB, UK

## 

The majority of experimental studies of synaptic plasticity in the brain have focussed on modifiable connections between excitatory neurons. However, recent developments in the area of inhibitory synaptic plasticity are challenging the view that inhibitory interneurons provide a purely regulatory role [1]. One such example is the observation that endocannabinoid mediated plasticity in GABAergic synapses may be source and target specific (both in terms of identity and location of pre and postsynaptic neurons), allowing for the suppression of inhibition in specific cortical layers and cell types [2]. Moreover, it has recently been shown that inhibitory synaptic plasticity can help balance excitation and inhibition and support the storage and retrieval of memories in recurrent neural networks [3].

In the present study, we explore the effect of different types of synaptic plasticity and other neuronal characteristics on memory performance in neural networks with excitatory and inhibitory subpopulations. In an initial set of simulations, we investigate the performance of networks of perceptrons with a variety of permitted synaptic plasticity regimes. We then extend this work using similarly configured networks of Izhikevich neurons, and we study how the capacity of these networks is affected by synaptic parameters and neuronal firing patterns.

The networks are trained using a modified version of the perceptron learning rule that enforces sign constraints, allowing model neurons to be labelled as excitatory or inhibitory. The retrieval of memories is tested by setting the initial state of the network to a corrupted version of the training pattern. In the non-spiking networks the network is allowed to settle into a steady state. In the networks of spiking neurons, we run the simulation for a fixed period of time and measure the performance by moving a sliding window across the firing trace to produce a set of firing vectors. These are compared to the uncorrupted training pattern and the firing vector with the highest similarity is selected. This process is carried out under increasing load (numbers of stored patterns) until the mean similarity across all tested patterns at a particular load *p* is < 95%. The *effective capacity* (the performance metric) is then said to be *p* – 1.

Although a sufficient degree of synaptic plasticity in the networks is required for them to act as associative memories, we find that a lack of plasticity can be tolerated in subsets of synapses. In particular, networks without plasticity at inhibitory-to-inhibitory synapses still perform well, with an effective capacity that is influenced by the weight of the non-plastic connections. Moreover, in the networks of spiking neurons the performance is strongly dependent on synaptic parameters such as decay and scaling, the types of neurons used in the inhibitory and excitatory subpopulations (such as regular or fast spiking neurons), and the sparsity of the training patterns.
